# Suitability of Capillary Blood for Quantitative Assessment of G6PD Activity and Performances of G6PD Point-of-Care Tests

**DOI:** 10.4269/ajtmh.14-0696

**Published:** 2015-04-01

**Authors:** Germana Bancone, Cindy S. Chu, Nongnud Chowwiwat, Raweewan Somsakchaicharoen, Pornpimon Wilaisrisak, Prakaykaew Charunwatthana, Pooja Bansil, Sarah McGray, Gonzalo J. Domingo, François H. Nosten

**Affiliations:** Shoklo Malaria Research Unit, Mahidol–Oxford Tropical Medicine Research Unit, Faculty of Tropical Medicine, Mahidol University, Mae Sot, Thailand; Mahidol–Oxford Tropical Medicine Research Unit, Faculty of Tropical Medicine, Mahidol University, Bangkok, Thailand; Diagnostics, PATH, Seattle, Washington; Centre for Tropical Medicine, Nuffield Department of Medicine, University of Oxford, Oxford, United Kingdom

## Abstract

The use of primaquine and other 8-aminoquinolines for malaria elimination is hampered by, among other factors, the limited availability of point-of-care tests for the diagnosis of glucose-6-phosphate dehydrogenase (G6PD) deficiency. Historically, the most used source of blood for G6PD analyses is venous blood, whereas diagnostic devices used in the field require the use of capillary blood; data have shown that the two sources of blood often differ with respect to hemoglobin concentration and number of red blood cells. Therefore, we have analyzed, in both capillary and venous blood drawn from the same healthy donors, the correlation of G6PD activity assessed by two qualitative tests (the Fluorescent Spot test and the CareStart test) with the gold standard quantitative spectrophotometric assay. Results obtained on 150 subjects with normal, intermediate, and deficient G6PD phenotypes show that, although differences exist between the aforementioned characteristics in capillary and venous blood, these do not impact on the quantitative assessment of G6PD activity after corrected for hemoglobin concentration or red blood cell count. Furthermore, we have assessed the sensitivity and specificity of the two qualitative tests against the gold standard spectrophotometric assay at different activity thresholds of residual enzymatic activity in both blood sources.

## Introduction

The widespread availability of 8-aminoquinoline, such as primaquine and in the future, tafenoquine, is critical in malaria elimination efforts in areas where *Plasmodium vivax* is prevalent. At present, only 8-aminoquinoline drugs can prevent relapse and eliminate the reservoir of hypnozoites in *P. vivax* infections; furthermore, they have gametocytocidal activity and are able to interrupt transmission to the invertebrate vector. However, individuals with G6PD deficiency are at risk of drug-induced hemolysis and can develop hemolytic anemia after exposure to the regimen used to eliminate the liver stages of *P. vivax*. For this reason, patients with G6PD deficiency should not be prescribed 8-aminoquinoline–based drugs.

The X-linked G6PD gene is perhaps the most polymorphic gene in the human genome. Because of selective pressure, G6PD mutations resulting in reduced erythrocytic G6PD activity levels are typically frequent in malaria-endemic regions.^1^ Thailand and southeast Asia are no exceptions, with G6PD deficiency reaching over 15% prevalence in certain populations.^2^,^3^ It is, therefore, critical to diagnose whether a patient is G6PD-deficient before administering an 8-aminoquinoline drug for radical cure of *P. vivax* malaria.

Target populations are often located in remote rural settings involving migratory populations, where antimalarial drugs are required, and point-of-care G6PD tests are needed to support broader availability of primaquine and in the future, tafenoquine. Although there are several commercially available G6PD tests, the large majority requires a cold chain for reagents and electricity to perform or read the test (Fluorescent Spot test [FST], Hirono, and WST-8/1-methoxy PMS).^4^–^6^ Furthermore, for the few tests that have undergone laboratory validation, namely the BinaxNOW (Alere Inc., Waltham, MA) and the CareStart (AccessBio, Somerset, NJ), the matrix choice has been venous blood.^7^,^8^ Previous studies suggest that several complete blood count (CBC) parameters, including white blood cell (WBC) count, red blood cell (RBC) count, and hemoglobin (Hb) levels, are higher in capillary blood compared with paired venous blood values.^9^–^11^ Without measuring the G6PD activity in paired capillary and venous blood, it is difficult to predict how the intrinsic differences in the two blood specimens will impact on the diagnosis of deficiency.

This study analyzed the correlation between G6PD activities (using the quantitative “gold standard” spectrophotometric assay) in venous and capillary blood in a sample of healthy volunteers with a broad range of enzymatic phenotypes. The performances of two qualitative tests (the FST and a point-of-care test) using capillary and venous blood were compared.

## Materials and Methods

### Study site and population.

This study was conducted at the Shoklo Malaria Research Unit (SMRU) in Mae Sot, Thailand along the Thai–Myanmar border (Tak Province); the clinical site mainly serves a migrant population composed of Burman and Karen ethnic groups. Healthy volunteers were recruited, and blood was collected at the clinics; samples were refrigerated for a maximum of 6 hours and shipped in cool boxes to the central hematology laboratory at the SMRU (in Mae Sot), where research procedures were conducted.

### Study design.

To calculate the sample size for the primary endpoint, we used an estimated expected correlation of 0.85 and set our desired precision at 0.1. This calculation resulted in 139 subjects needed to achieve the primary objective of measuring correlation between capillary and venous blood results using the quantitative spectrophotometric G6PD assay as the reference. To achieve the secondary endpoints (concordance between a qualitative G6PD test and the spectrophotometric assay and measure of the categorical accuracy of a qualitative G6PD test against the spectrophotometric assay), the total sample size was increased to 150 patients. A targeted enrollment was used to achieve a convenience sample of approximately 50 G6PD-deficient volunteers (male and female), approximately 50 G6PD-heterozygous female volunteers, and G6PD-normal volunteers with a 1:1 male-to-female ratio. A specific protocol for searching the clinical database of the SMRU was set up, allowing the researchers to compile a list of possible volunteers with a known G6PD phenotype or genotype; the study staff linked the unique identifier code provided by the researcher with the demographic data and organized home visits to invite the subjects to the study. The day of the appointment at the clinic, the volunteers went through the normal consent process, including full explanation of the study, signature of informed consent, and blood sampling.

### Ethical considerations.

Ethical approvals for this study were obtained from the Mahidol University Faculty of Tropical Medicine Ethics Committee (FTMEC), the Oxford Tropical Research Ethics Committee (OxTREC), and the PATH Research Ethics Committee (REC). The protocol was also reviewed by the Community Advisory Board at SMRU, which is composed of representatives from the communities served by SMRU. Volunteers who met the inclusion criteria underwent a detailed informed consent process and provided written consent before enrolling in the study.

### Blood sampling.

Two milliliters venous blood was drawn from the arm, transferred to a K2 ethylenediaminetetraacetic acid (EDTA) tube (BD Vacutainer, Franklin Lakes, NJ), and inverted 10 times; 100 μL capillary blood was drawn by the finger using a standard lancet and collected in EDTA SAFE-T-FILL capillary tubes (Kabe Labortechnik, Nümbrecht-Elsenroth, Germany). All samples were kept at 4°C until analysis.

### Laboratory procedures.

Two different qualitative tests and one quantitative spectrophotometric assay for G6PD were performed on both capillary and venous blood samples together with a CBC.

The G6PD FST (R&D Diagnostic, Aghia Paraskevi, Greece) was performed using 5 μL blood mixed with 100 μL reagents; after 10 minutes of incubation at room temperature, a 15-μL aliquot was spotted on filter paper and allowed to air dry. The spots were then visualized under ultraviolet (UV) light; spots that showed fluorescence were classified as normal, and spots that failed to show fluorescence were classified as deficient.

The CareStart test (AccessBio) was performed according to the manufacturer's instructions: 2 μL blood was placed in the device, and the buffer was added immediately; after 10 minutes, the reading window was inspected for development of color. Tests showing a pink color were classified as normal, tests showing no color were classified as deficient, and tests that showed remaining blood in the reading window were considered invalid.

The G6PD spectrophotometric assay (Trinity Biotech, Bray, Ireland) was performed in duplicate using 10 μL whole blood per replicate; instructions from the supplier were followed for reagents preparation. A UV-1800 (SHIMADZU Corporation, Kyoto, Japan) spectrophotometer with an electronically controlled temperature compartment was used to detect the absorbance at 340 nm for 10 minutes at 30°C. G6PD activity was calculated as international units per gram Hb and units per RBC using the results of the CBC on the same blood.

The CBC was performed using a CeltacF MEK-8222K hematology analyzer (Nihon Kohden, Tokyo, Japan). The CBC included WBC (total and differential count), RBC count, RBC size (mean cell volume [MCV]), Hb content (mean corpuscular hemoglboin [MCH] and mean corpuscular hemoglobin concentration [MCHC]), total Hb concentration (HGB), hematocrit (HCT), and platelet count (PCT). Reproducibility of selected features is detailed in Table 1. Quality controls were run every day before analysis of samples.

### Statistical methods.

To examine the relationship between capillary and venous blood, data from the quantitative spectrophotometric assays adjusted for RBC (units per RBC) and Hb (international units per gram Hb) were plotted, and a linear regression was used to calculate the unstandardized coefficients and standard error (*B* ± SE) indicating the change in G6PD activity in venous blood when G6PD activity changes in capillary blood. Bland–Altman analyses were conducted to visually assess the agreement between the test results. The mean differences, SD of the difference, and a 95% tolerance-bound mean difference ± 1.96 SD (limits of agreement) were calculated and plotted. The percentage agreement between capillary and venous blood for the CareStart test and the FST was calculated and compared using Cohen's κ-coefficient to measure concordance between the two tests. For this analysis, the CareStart test results that were invalid were excluded.

The G6PD quantitative spectrophotometric assay from capillary and venous blood was used as the reference assay; the G6PD median value for normal males was calculated using the data from 26 G6PD-normal males, and the respective 20%, 30%, and 40% thresholds were used to assess performances of qualitative tests. The clinical performances (sensitivity, specificity, positive predictive value [PPV], and negative predictive value [NPV]) of both the CareStart test and the FST for G6PD deficiency were evaluated for each of the cutoff points. All statistical analyses were conducted in Stata 12.0 (Statacorp, College Station, TX).

## Results

### Hematologic features of capillary and venous blood.

The analysis of correlation between capillary and venous blood samples from the same volunteer confirms that the two blood sources differ slightly for the number of RBCs and Hb concentration. Table 2 compares a few relevant hematologic features between capillary and venous blood, and it shows that Hb concentration and number of RBCs are higher in capillary blood compared with venous blood.

### Quantitative spectrophotometric assay.

For the analysis of the enzymatic phenotype across the different blood sources, the activity was calculated adjusting for Hb concentration (as international units per gram Hb), RBC count (as units per RBC), or uncorrected difference in absorbance over 10 minutes (ΔAbs).

**Figure 1.**
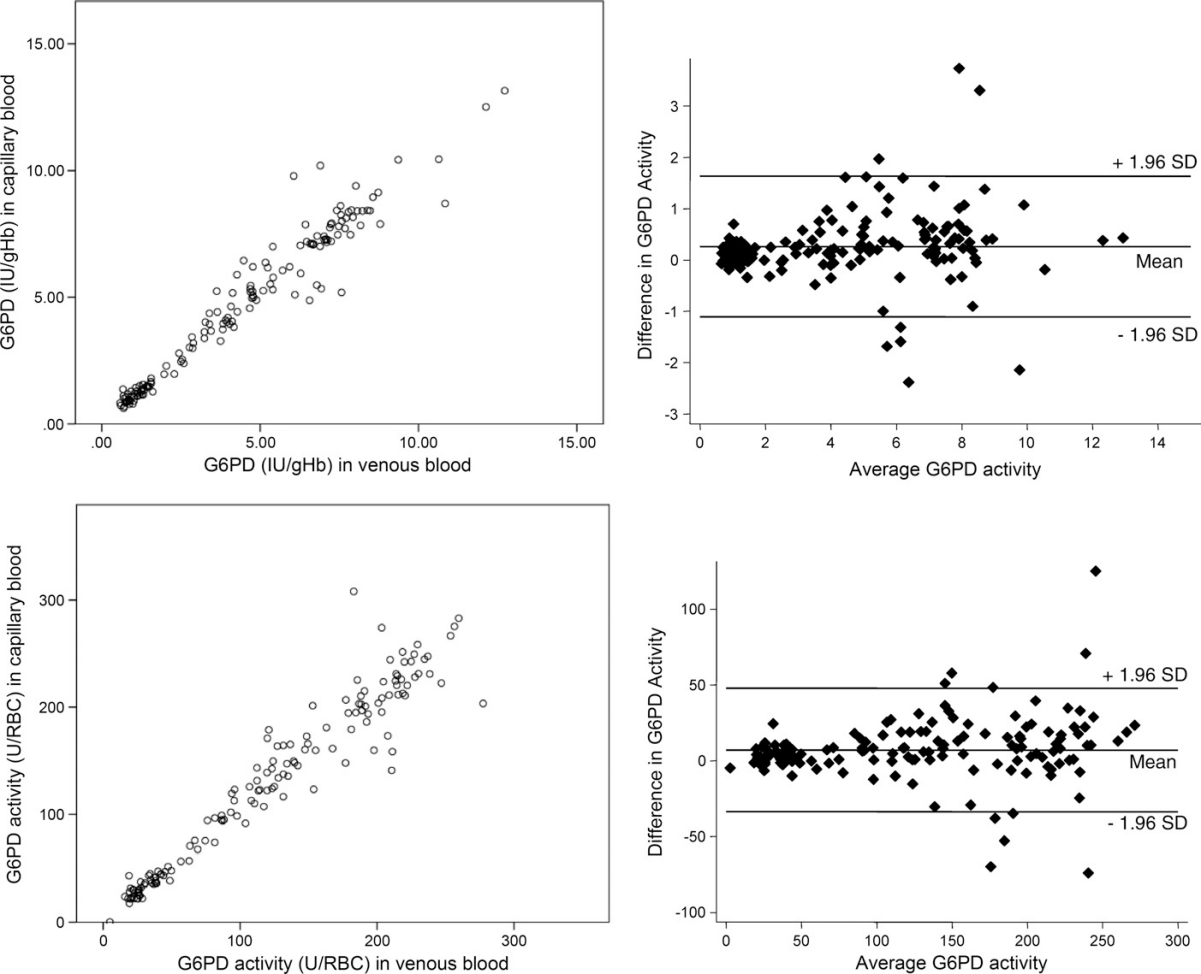
Comparisons of G6PD enzymatic activities across blood sources. (Upper Left) Dot plot and (Upper Right) Bland–Altman plot of G6PD activities expressed as international units per gram Hb in paired samples of capillary and venous blood. (Lower Left) Dot plot and (Lower Right) Bland–Altman plot of G6PD activities expressed as units per RBC in paired samples of capillary and venous blood.

The data show a high correlation in the enzymatic activity from capillary and venous blood estimated with the two approaches (Figure 1). G6PD activity expressed as international units per gram Hb has a *B* ± SE = 1.011 ± 0.020 between capillary and venous blood; G6PD activity expressed as units per RBC has a *B* ± SE = 1.009 ± 0.022 between the two blood sources. The absorbance raw data (uncorrected for RBCs or Hb concentration) show a lower correlation between capillary and venous blood sources with a *B* ± SE = 1.075 ± 0.014.

### Agreement in the diagnosis of deficient subjects across the two sources of blood for the qualitative tests.

Table 3 shows the results of the two qualitative tests: the CareStart test and the FST. In the analysis of the CareStart test, 13 (*N* = 13) samples with invalid test results were excluded. The percentage agreement between capillary and venous blood samples for the CareStart test was 89.05% (κ= 0.7565, *P* value < 0.01). The percentage agreement between capillary and venous blood for the FST was 98.67% (κ= 0.9694, *P* value < 0.01).

### Performance of the CareStart test and the FST against the spectrophotometric assay.

The normal reference values for the spectrophotometric assay for the two types of samples were calculated in 26 normal males and are shown in Table 4.

The sensitivity, specificity, PPV, and NPV for the CareStart test and the FST were calculated according to different thresholds of activity expressed as percentages of the normal activity in the specific type of sample source; Tables 5 and 6 show the results for three selected thresholds according to the Hb-normalized (international units per gram Hb) and uncorrected activity results.

## Discussion

### Comparisons of G6PD activities in paired samples of capillary and venous blood.

The diagnosis of G6PD deficiency in the field is usually performed with capillary blood (fresh or spotted on filter paper) using different methodologies (FST, Hirono, or WST8/1-methoxy PMS).^4^–^6^ Quantitative assessment of G6PD is, instead, more often performed on venous blood for several reasons, including volume requirement (the World Health Organization standard protocol) and ease of blood sampling and storage.^12^ The two sampling methods also often use different anticoagulants (EDTA for venous blood and heparin for capillary sampling). Because some data have shown differences in various hematologic features between the two sources of blood in both healthy subjects and patients of different ages, a sensible strategy for the validation and deployment of point-of-care tests requires a comparison of suitability of sources of blood (capillary and venous) followed by field validation of new tests against the gold standard in the chosen blood type (capillary).^9^–^11^

Hematologic features of capillary and venous blood relevant for the diagnosis of G6PD are the number of RBCs, the HGB, and the HCT. The number of WBCs is also thought to influence the total G6PD activity if whole blood is used, but large variations between blood sources are not expected in healthy volunteers. Furthermore, G6PD activity is conventionally expressed as international units per gram Hb, and in populations where anemia is common, the resulting estimation of enzymatic activity might be falsely increased by a low concentration of Hb in the blood. A less commonly used but reliable alternative estimation is based on calculation of the enzymatic activity as units per RBC. Although this study only included healthy volunteers and no malaria infections were found, almost 20% of study subjects had an Hb below 12 g/dL, allowing for a comparison of G6PD activity expressed in both ways.

The data from this study on capillary and venous blood collected on the same healthy volunteers confirmed the previous findings that capillary blood had a higher number of RBCs and higher concentrations of Hb and HCT.^9^ WBCs count and mean corpuscular volume were not significantly different, whereas platelets were lower in capillary blood compared with venous blood. The differences found in the two blood sources were less than 5%, and they were not expected to be of clinical relevance. Our data showed that these differences did not have an impact on the assessment of enzymatic activity using the spectrophotometric assay. There was a highly significant correlation between the enzymatic activities assessed in the two different sources of blood when activity was expressed as international units per gram Hb or units per RBC. The correlation between G6PD activity in venous versus capillary blood was lower when it was not normalized by Hb or number of RBCs (i.e., expressed as raw difference in absorbance).

### Clinical performances of the CareStart test and the FST.

A premise to the interpretation of the results of performance of the two qualitative tests is that, in heterozygous females, the proportion of RBCs with a functional G6PD protein is unpredictable and varies greatly among subjects with the same genotype. Therefore G6PD phenotypic methodologies do not have the power to identify the genotype of the tested females; as a consequence, analyzing the sensitivity and specificity of qualitative tests with respect to the genotype is methodologically incorrect when heterozygous females are included in the analyses. The performances of new qualitative phenotypic tests should be assessed by either analyzing only samples from males or when females are included, comparing with a quantitative assessment of activity performed using the gold standard quantitative method (which is the spectrophotometric assay).^12^ In this study, we have used the second approach.

The FST has been in use for more than 40 years, but data about its performances compared with spectrophotometry are scarce.^4^ A large study performed among over 1.2 million Greek newborns using blood collected on filter paper estimated that the detection threshold of the FST was around 20% of normal activity.^13^ When females were included in screenings using the FST, many studies reported a low performance of the qualitative test compared with genotyping, with a low assessed sensitivity (lower than 45% in some studies); in a few studies, a specific intermediate phenotype had to be added to the results to increase sensitivity.^14^–^17^ A recent laboratory-based validation of the FST showed rather different performances in sensitivity and specificity when the intermediate phenotype was included in the deficient or the normal group.^18^ In a recent study performed in Uganda, of 461 malaria patients classified as G6PD-normal by the FST, 27 (5.9%) were found to be homozygous/hemizygous, and 61 were heterozygous (13.2%) for G6PD variant A−.^19^ It is unclear whether this was caused by a higher enzymatic activity associated with the African variant in that population or more likely, the medical condition (acute malaria infection) of the subjects under study. Because the test is used commonly on cord blood for newborn screening, the higher number of reticulocytes is also expected to increase the number of false-normal results.^20^ Previous data collected in the SMRU in the last 4 years showed that the test was reliable, even when performed in field-based simple laboratories.^21^ Nonetheless, its main limitation are the electricity requirement for cold-chain reagent storage and test reading, which has prevented its field use in more remote areas.

The G6PD CareStart test has been used in its first formulation in a large trial in Cambodia; the characteristics of heat and humidity stability for both storage and test performances were excellent but the test showed an alarmingly low sensitivity (68%) in the detection of G6PD-deficient subjects.^8^ More recently, in Haiti, its sensitivity for detecting deficient subjects with activity below 30% of normal was estimated to be 84.8%.^22^ Although von Fricken and others^22^ consider this a “highly sensitive” test, it has to be noted that it would diagnose as G6PD-normal more than 15% of subjects with a very low residual enzymatic activity.

From a clinical perspective, it is difficult to establish at which threshold of enzymatic activity the ideal point-of-care test should be set, because data are not yet available on the precise correlation between the patients' enzymatic activity and the hemolytic risk at a given drug dosage. It is commonly accepted that subjects with enzymatic activity below 30% of the normal are at greater risk of clinically severe hemolysis if treated with primaquine for radical cure of *P. vivax* malaria. In this study, we chose to analyze the performances of the two qualitative tests at three different relevant thresholds, namely 20%, 30%, and 40% activity. Furthermore, the performances of the qualitative tests were analyzed with respect to both the corrected activity (expressed as international units per gram Hb) and the uncorrected absorbance.

Considering the mentioned thresholds, the FST performed well on venous blood, with a higher sensitivity and specificity at each of the thresholds compared with the CareStart test. This could also explain why the CareStart test showed a level of agreement slightly below 90% in the diagnosis of deficiency across the different sources of blood, whereas FST showed a very good agreement (98.6%). Both tests showed a comparable or slightly lower sensitivity against the uncorrected activity compared with the activity expressed in international units per gram Hb. This seemed to indicate that the visual result of both tests, in terms of fluorescence or color development, might be influenced by the Hb content of the blood sample. As a confirmation, in the experience with the FST in the hematology laboratory of the SMRU, it has been noted before that samples from G6PD-normal anemic subjects (especially pregnant women) do tend to show a brighter fluorescence compared with G6PD-normal subjects without anemia.

Results on the capillary blood showed that the CareStart test had a higher sensitivity but lower specificity compared with the FST at all thresholds. We noted that, with regard to capillary blood, the CareStart test had a very high rate (almost 10%) of invalid results.

In summary, the new point-of-care test (CareStart), although promising and with appropriate characteristics for use in remote field conditions, did not perform as well as the classic FST on venous blood. In capillary blood, although the ability to detect deficient samples increased, the number of invalid results also increased.

## Conclusions

The small differences in hematologic parameters of capillary and venous blood did not influence the assessment of G6PD activity using the quantitative spectrophotometric assay; therefore, capillary blood collected from finger prick can be considered suitable for quantitative assessment of G6PD activity and diagnosis of G6PD deficiency. The performances of two qualitative tests for the rapid diagnosis of G6PD deficiency in the field were compared in both sources of blood with the gold standard. The FST showed a very good agreement in the diagnosis of G6PD-deficient subjects in the two types of sample (98.6%), whereas the CareStart test had a level of agreement below 90%. Clinical performances of both tests in the two blood sources showed a high sensitivity at the 20% and 30% activity thresholds, with an assessed sensitivity for severely deficient subjects ranging from 89.1% to 82.5% (CareStart) and 95.7% to 97.6% (FST) in venous blood and over 97.5% in both tests in capillary blood. More studies should be performed on the CareStart test, because the test, in the formulation tested during this study, showed a high rate (almost 10%) of invalid results in capillary blood samples.

## Figures and Tables

**Table 1:** Technical characteristics of the automated hematology analyzer CeltacF MEK-8222K

Measured parameters	Measuring range	Reproducibility to specimen from venous blood
WBC	Count 0–599 × 10^3^/μL	Within 2.0% CV (4.0–9.0 × 10^3^/μL)
RBC	Count 0–14.9 × 10^6^/μL	Within 1.5% CV (5.0 × 10^6^/μL)
HGB	0–29.9 g/dL	Within 1.5% CV (16 g/dL)
HCT	0–99.9%	
MCV	20–199 fL	Within 1.0% CV (70–120 fL)
PCT	0–1,490 × 10^3^/μL	Within 4.0% CV (3.0 × 10^3^/μL)

CV = coefficient of variation.

**Table 2:** Comparison of hematologic features (mean [SD]) of capillary and venous blood samples collected in the study

Blood source	*N*	WBC (10^3^/μL)	RBC (10^6^/μL)	Hb (g/dL)	HCT (%)	MCV (fL)	PCT (10^3^/μL)
Capillary	150	6.48 (1.95)	4.78 (0.71)	13.38 (1.70)	40.03 (5.06)	84.24 (7.03)	275.56 (79.55)
Venous	150	6.51 (1.98)	4.62 (0.65)	12.91 (1.64)	38.34 (4.71)	83.45 (6.74)	296.67 (83.36)
*P*_(ANOVA)_		0.916	0.039	0.015	0.003	0.321	0.026
*P*_paired *t* test_		0.705	< 0.001	< 0.001	< 0.001	< 0.001	< 0.001
Difference (%; capillary − venous)		−0.04	3.4	3.5	4.2	0.9	−7.7

ANOVA = analysis of variance.

**Table 3:** Summary results of the CareStart test and the FST on the two blood sources

Capillary	Venous		
	Normal	Deficient	Total
CareStart			
Normal	84	0	84
Deficient	15	38	53
Total	99	38	137*
FST			
Normal	101	0	101
Deficient	2	47	49
Total	103	47	150

* Invalid results (13) were excluded from analysis.

**Table 4:** Median reference values of G6PD enzymatic activity in the two blood sources

Blood source	Activity (IU/g Hb)	Activity (U/RBC)	Uncorrected (ΔAbs in 10 minutes)
Venous	7.51	209.28	0.220
Capillary	7.61	209.43	0.233

ΔAbs = change in absorbance.

**Table 5:** Clinical performances of the CareStart test and the FST on venous blood for detection of deficient G6PD activity at three thresholds

	Activity (IU/g Hb)			Uncorrected ΔAbs		
	20% Activity threshold	30% Activity threshold	40% Activity threshold	20% ΔAbs threshold	30% ΔAbs threshold	40% ΔAbs threshold
Number of G6PD-deficient	41	47	56	44	53	65
Sensitivity (%; 95% CI)						
CareStart test*	92.5 (79.6–98.4)	89.1 (76.4–96.4)	76.4 (63.0–86.8)	93.0 (80.9–98.5)	80.8 (67.5–90.4)	65.6 (52.7–77.1)
FST^†^	97.6 (87.1–99.9)	95.7 (85.5–99.5)	83.9 (71.7–92.4)	97.7 (88.0–99.9)	88.7 (77.0–95.7)	72.3 (59.8–82.7)
Specificity (%; 95% CI)						
CareStart test*	94.5 (88.4–98.0)	98.1 (93.2–99.8)	98.9 (94.2–100.0)	97.2 (92.0–99.4)	99.0 (94.4–100.0)	98.8 (93.6–100.0)
FST†	93.6 (87.2–97.4)	98.1 (93.2–99.8)	100.0 (96.2–100.0)	96.2 (90.6–99.0)	100.0 (94.4–100.0)	100.0 (93.7–100.0)
PPV (%; 95% CI)						
CareStart test*	86.0 (72.1–94.7)	95.3 (84.2–99.4)	97.7 (87.7–99.9)	93.0 (80.9–98.5)	97.7 (87.7–99.9)	97.7 (87.7–99.9)
FST†	85.1 (71.7–93.8)	95.7 (85.5–99.5)	100.0 (92.5–100.0)	91.5 (79.6–97.6)	100.0 (988.9–100.0)	100.0 (88.9–100.0)
NPV (%; 95% CI)						
CareStart test*	97.2 (92.0–99.4)	95.3 (89.3–98.5)	87.7 (79.9–93.3)	97.2 (92.0–99.4)	90.6 (83.3–95.4)	79.2 (70.3–86.5)
FST†	99.0 (94.7–100.0)	98.1 (93.2–99.8)	91.3 (84.1–95.9)	99.0 (94.7–100.0)	94.2 (87.8–97.8)	82.5 (73.8–89.3)

ΔAbs = change in absorbance; 95% CI = 95% confidence interval.

* Clinical performance estimated using 149 participants (1 participant had an invalid CareStart test result).

† Clinical performance estimated using 150 participants.

**Table 6:** Clinical performance of the CareStart test and the FST on capillary blood for detection of deficient G6PD activity at three thresholds

	Activity (IU/g Hb)			Uncorrected ΔAbs		
	20% Activity threshold	30% Activity threshold	40% Activity threshold	20% Activity threshold	30% Activity threshold	40% Activity threshold
Number of G6PD-deficient	40	47	54	42	50	57
Sensitivity (%; 95% CI)						
CareStart test*	100.0 (90.0–100.0)	100.0 (91.4–100.0)	95.7 (85.5–99.5)	100 (86.5–100.0)	97.7 (88.0–99.9)	96.0 (86.3–99.5)
FST†	97.5 (86.8–99.9)	97.9 (88.7–99.9)	87.0 (75.1–94.6)	100 (87.7–100.0)	96.0 (86.3–99.5)	86.0 (74.2–93.7)
Specificity (%; 95% CI)						
CareStart test*	81.6 (72.7–88.5)	86.6 (78.2–92.7)	90.1 (82.1–95.4)	84.0 (75.3–90.6)	88.3 (80.0–94.0)	93.2 (85.7–97.5)
FST†	92.7 (86.2–96.8)	99.0 (94.7–100.0)	100.0 (96.2–100.0)	93.5 (87.1–97.4)	99.0 (94.6–100.0)	100 (94.2–100.0)
PPV (%; 95% CI)						
CareStart test*	64.8 (50.6–77.3)	75.9 (62.4–86.5)	83.3 (70.7–92.1)	70.4 (56.4–82.0)	79.6 (66.5–89.4)	88.9 (77.4–95.8)
FST†	83.0 (69.2–92.4)	97.9 (88.7–99.9)	100.0 (92.5–100.0)	85.7 (72.8–94.1)	98.0 (89.1–99.9)	100 (89.4–100.0)
NPV (%; 95% CI)						
CareStart test*	100.0 (95.7–100.0)	100.0 (95.7–100.0)	97.6 (91.7–99.7)	100 (93.6–100.0)	98.8 (93.5–100.0)	97.6 (91.7–99.7)
FST†	99.0 (94.7–100.0)	99.0 (94.7–100.0)	93.2 (86.5–97.2)	100 (94.7–100.0)	98.0 (93.0–99.8)	92.1 (85.0–96.5)

ΔAbs = change in absorbance; 95% CI = 95% confidence interval.

* Clinical performance estimated using 137 participants; 13 participants had an invalid CareStart test result.

† Clinical performance estimated using 150 participants.
